# Advanced Molecular Electron Density Theory Study of the Substituent Effects in Nucleophilic Substitution Reactions

**DOI:** 10.1021/acsomega.5c00957

**Published:** 2025-07-07

**Authors:** Luis R. Domingo, Patricia Pérez, Mar Ríos-Gutiérrez, María José Aurell

**Affiliations:** † Department of Organic Chemistry, University of Valencia, Dr. Moliner 50, 46100 Burjassot, Valencia, Spain; ‡ Avd. Tirso de Molina 20, 46015 Valencia, Spain; § Facultad de Ciencias, Campus Ciudad Universitaria, 28088 Universidad San Sebastián, Av. del Condor 720, Ciudad Empresarial, Huechuraba, Santiago 8580704, Chile

## Abstract

The energetic and structural effects of the CH_3_, CH=CH_2_, Ph, CHO, and OCH_3_ groups present on the tetrahedral carbon involved in nucleophilic substitution (SN) reactions of primary substituted carbons have been studied within the molecular electron density theory (MEDT). Electron localization function analysis at the ground state indicates no remarkable electronic changes in the tetrahedral carbon due to substitution. The low electrophilic character of the substrates suggests that they lack the propensity to react with nucleophiles. The activation energies of the three selected series of SN reactions, which include the participation of a strong nucleophile and a very good leaving group (LG), range from 0.3 to 23.5 kcal·mol^–1^, decreasing in each series in the order CH_3_ > H > CH=CH_2_ ≈ CHO > Ph > OCH_3_, with the last group being highly activating. A relative interacting atomic energy analysis of the transition state structures involved in the series of symmetric SN reactions involving chloride anion Cl^–^ allows for an understanding of the electronic effects of these groups on the kinetics of these reactions. The present MEDT study emphasizes that both the electronic effects of the groups present on the primary substituted tetrahedral carbon and the nature of the LG can shift the molecular mechanism of the SN reactions from an S_N_2 to an S_N_1 one.

## Graphical abstract

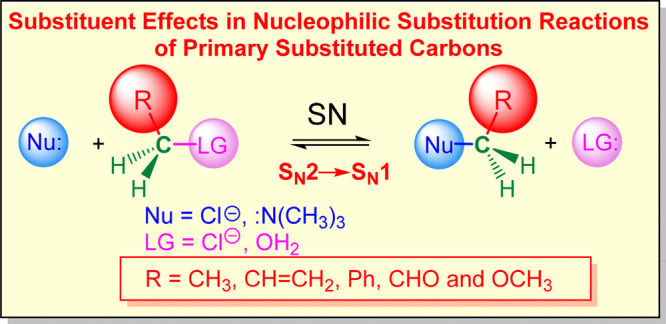

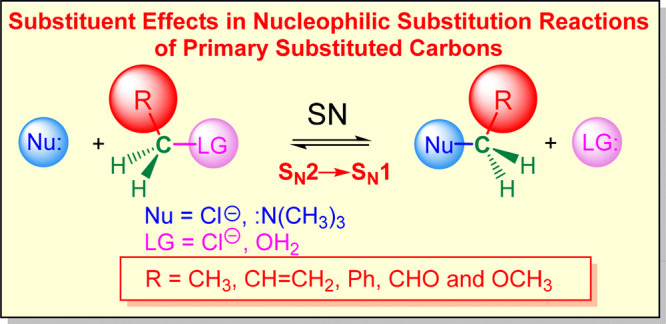

## 1 Introduction

Nucleophilic substitution (SN) reactions, developed by Ingold and Hughes in the 1930s,^1–3^ are fundamental reactions of broad synthetic and academic interest. In general, in an SN reaction, an atom or a group of atoms called the leaving group (LG), attached to a tetrahedral carbon, is replaced by an atom or a group of atoms called the nucleophile (Nu). Various combinations of charged and uncharged species participate in SN reactions (see Scheme 1).^4^

**Scheme 1:**
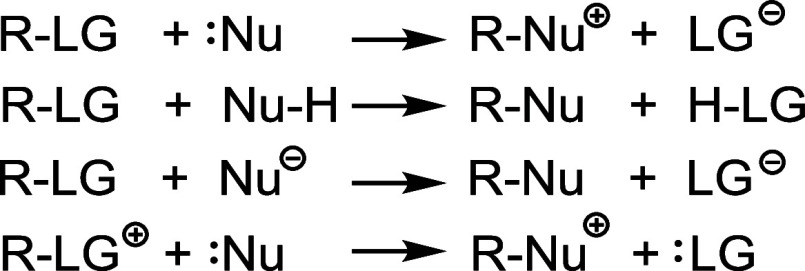
Most Common SN Reaction Types Involving Charged and Uncharged Species

Hughes and Ingold^1^ defined two limiting mechanisms: unimolecular nucleophilic substitution (S_N_1) and bimolecular nucleophilic substitution (S_N_2) (see Scheme 2). While a stepwise mechanism involving the formation of a carbocation intermediate in the rate-determining step was proposed for the S_N_1 reaction, a concerted mechanism via a single transition state structure (TS) involving a pentacoordinate carbon^4^ has been proposed for the S_N_2 reaction (see Scheme 2).

**Scheme 2:**
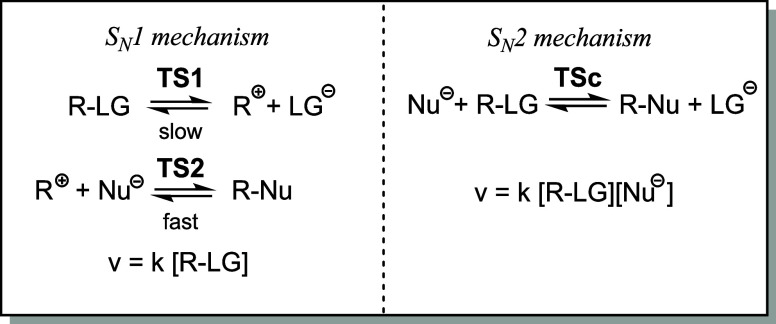
Two Proposed Limiting Mechanisms, S_N_1 and S_N_2, for the Nucleophilic Substitution Reactions

The SN reactions have been extensively studied over the years,^4,5^ with a particular focus on their theoretical understanding.^6–24^ Thus, several recent DFT-based studies have examined nucleophilic substitution mechanisms using energetic and orbital descriptors. For instance, Li and Xue^21^ investigated substituent effects in gas-phase S_N_2 reactions of phenoxides, while Hamlin et al.^22^ analyzed the effects of the central atom, nucleophile, and solvent. More recently, Kuo et al.^23^ explored nonclassical halogenophilic (SN_2_X) and chalcogenophilic (SN2Ch) substitution mechanisms using quantitative DFT descriptors. Although these approaches provide valuable energy profiles and orbital-based rationalizations, they often lack a localized description of electron density reorganization in the TSs. In this context, the use of the electron localization function^25^ (ELF) and the recently proposed relative interacting atomic energy^26^ (RIAE) analysis within the molecular electron density theory^27^ (MEDT) framework provides a unique perspective by permitting a topological and energetic decomposition of the evolution of electron density. Indeed, these methods reveal substituent-dependent changes in bonding regions and interfragment interactions, resulting in a deeper and more chemically intuitive understanding of the reactivity. Thus, in 2008, Andrés et al.^24^ reported a theoretical study based on ELF and catastrophe theory analyses of the forming/breaking bond processes and electronic rearrangements along the reaction path associated with the molecular mechanism for the gas-phase symmetric XCH_3_ + X^–^ (X = F, Cl, Br) S_N_2 reactions. This study highlighted that in these symmetric S_N_2 reactions the TSs were characterized by ionic species and lacked a disynaptic basin connecting the halogens and the central carbon atom.

The kinetics of SN reactions depend on several structural factors, such as the electronic nature of both LG and Nu, the substitution pattern of the tetrahedral carbon undergoing the SN reaction, and even solvent effects, which can stabilize ionic species.^4,5^

In 2006, Pérez et al.^28^ proposed a scale of nucleofugality for the LG participating in SN reactions, based on the electrophilicity ω index of the CH_3_–LG molecules computed at the B3LYP/6-31G­(d) computational level in the gas phase. While NH_2_, OH, or OCH_3_ were the worst LGs, the corresponding protonated species, such as OH_2_^+^ or OHCH_3_^+^, were the best. On this scale, N_2_^+^ LG was the best. The reactivity of the halogen atoms as LGs was correctly predicted; I > Br > Cl > F.^28^

Due to the relevance of adequately characterizing the TSs associated with SN reactions and thus understanding their molecular mechanisms from a theoretical point of view, the S_N_2 reactions of four monosubstituted methyl derivatives **1a,d**, that is, two symmetric and four nonsymmetric S_N_2 reactions, were recently studied within the MEDT (see Scheme 3).^29^

**Scheme 3:**
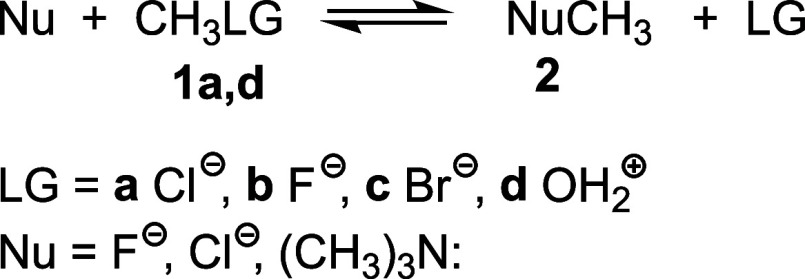
S_N_2 Reactions of the Monosubstituted Methyl Derivatives **1a,d**

Both ELF^25^ and quantum theory of atom-in-molecules^30,31^ (QTAIM) topological analyses of the electron density distribution at the TSs involved in these S_N_2 reactions indicated that they can be described as a central methyl carbocation, CH_3_^+^, strongly stabilized by the presence of two neighboring Nu and LG species through an electron density transfer process. This finding rejected the previously proposed hypervalent carbon atom at the TSs of the concerted S_N_2 reactions.^20^ Since SN reactions are reversible, the LG can be considered a Nu after its departure; in fact, in the symmetric S_N_2 reactions, the Nu and the LG are the same species.^29^ Except for protonated methanol CH_3_OH_2_^+^ **1d**, which is positively charged, the other methyl derivatives CH_3_X (X = F, Cl, or Br) **1a,c** presented electrophilicity ω indices below 0.87 eV, indicating the poor electrophilic character of these neutral halo methyl derivatives and, therefore, their low tendency to participate in polar reactions with nucleophiles. Consequently, the departure of LG is expected to occur before the Nu approach. This MEDT study established an electronic similarity between the molecular mechanisms of the so-called S_N_1 and S_N_2 reactions; the strong electronic stabilization of the tertiary carbocation, (CH_3_)_3_C^+^, does not demand the participation of Nu.^29^ Consequently, this SN reaction is associated only with the departure of the LG.

Herein, a MEDT study of the SN reactions of five monosubstituted chloromethanes **3a**–**e** and five protonated monosubstituted methanol derivatives **4a**–**e** is carried out to understand how the presence of CH_3_, CH=CH_2_, Ph, CHO and OCH_3_ groups at the tetrahedral carbon modifies structural and the energetic aspects, as well as the molecular mechanism of the corresponding SN reactions on primary substituted carbon compounds (see Scheme 4). Note that the 3-chloropropene **3b** and protonated prop-2-en-1-ol **4b** can undergo an S_N_2′ reaction, in which nucleophiles attack the methylene CH_2_ position of the CH=CH_2_ double bond, which is not considered herein.^19^ Three series of SN reactions involving the aforementioned five groups have been studied: (i) the symmetric SN reactions of the chlorine derivatives **3a**–**e** with the chlorine anion Cl^–^ as the Nu, series I; (ii) the SN reactions of the chlorine derivatives **3a**–**e** with the good Nu trimethyl amine (CH_3_)_3_N:, series II; finally, (iii) the SN reactions of the protonated alcohols **4a**–**e**, having the very good LG water H_2_O molecule, with chlorine anion Cl^–^ as Nu, series III (see Scheme 4).

**Scheme 4:**
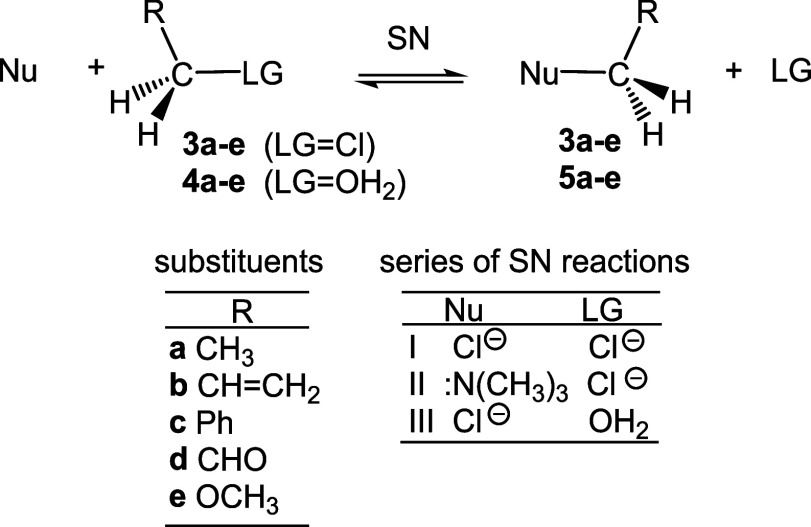
SN Reactions of Five Monosubstituted Chloromethanes **3a**–**e** and Five Protonated Monosubstituted Methanol Derivatives **4a**–**e**

Recently, the RIAE^26^ analysis has been introduced into the MEDT studies to analyze the electronic factors responsible for the activation energies of organic reactions. After an interacting quantum atom^32^ (IQA) energy decomposition analysis of the intra- and interatomic energies of the atoms, RIAE performs a comparative analysis of the changes in the corresponding atomic energies at the ground and the transition states to identify the electronic factors responsible for the activation energies. The theoretical background of the RIAE analysis is provided in the Supporting Information. Accordingly, an RIAE analysis is carried out on series I of symmetric SN reactions of chloromethyl-substituted compounds **3a**–**e** with the chlorine anion Cl^–^ to establish the role of the substituents in reducing the activation energies of these SN reactions from an electronic perspective.

## 2 Results and Discussion

The present MEDT study has been organized into five sections: (i) First, an ELF topological analysis of the ground-state (GS) electronic structure of disubstituted methyl compounds (DMCs) **3a**–**e** and **4a**–**e** is carried out; (ii) second, an analysis of the DFT-based reactivity indices at the GS of the reagents is performed; (iii) the third section explores the reaction paths associated with the SN reactions of DMCs **3a**–**e** and **4a**–**e**; (iv) the fourth section provides a comparative ELF topological analysis of the TSs associated with the SN reactions of DMCs **3a–e** and **4a–e**; finally, (v) the fifth section focuses on an RIAE analysis of the symmetric SN reactions of the chloromethyl-substituted compounds **3a**–**e** with chlorine anion Cl^–^.

### 2.1 ELF Analysis of the GS Electronic Structures of DMCs **3a**–**e** and **4a**–**e**

The topological analysis of the ELF^25^ allows for a quantitative characterization of the electron density distribution in a molecule,^33^ facilitating a correlation between its electronic structure and reactivity. As such, an ELF topological analysis of the electronic structures of DMCs **3a**–**e** and **4a**–**e**, used as the substrates in these SN reactions, was performed first. ELF basin attractor positions and the most relevant valence basin populations are listed in Figure 1.

**Figure 1:**
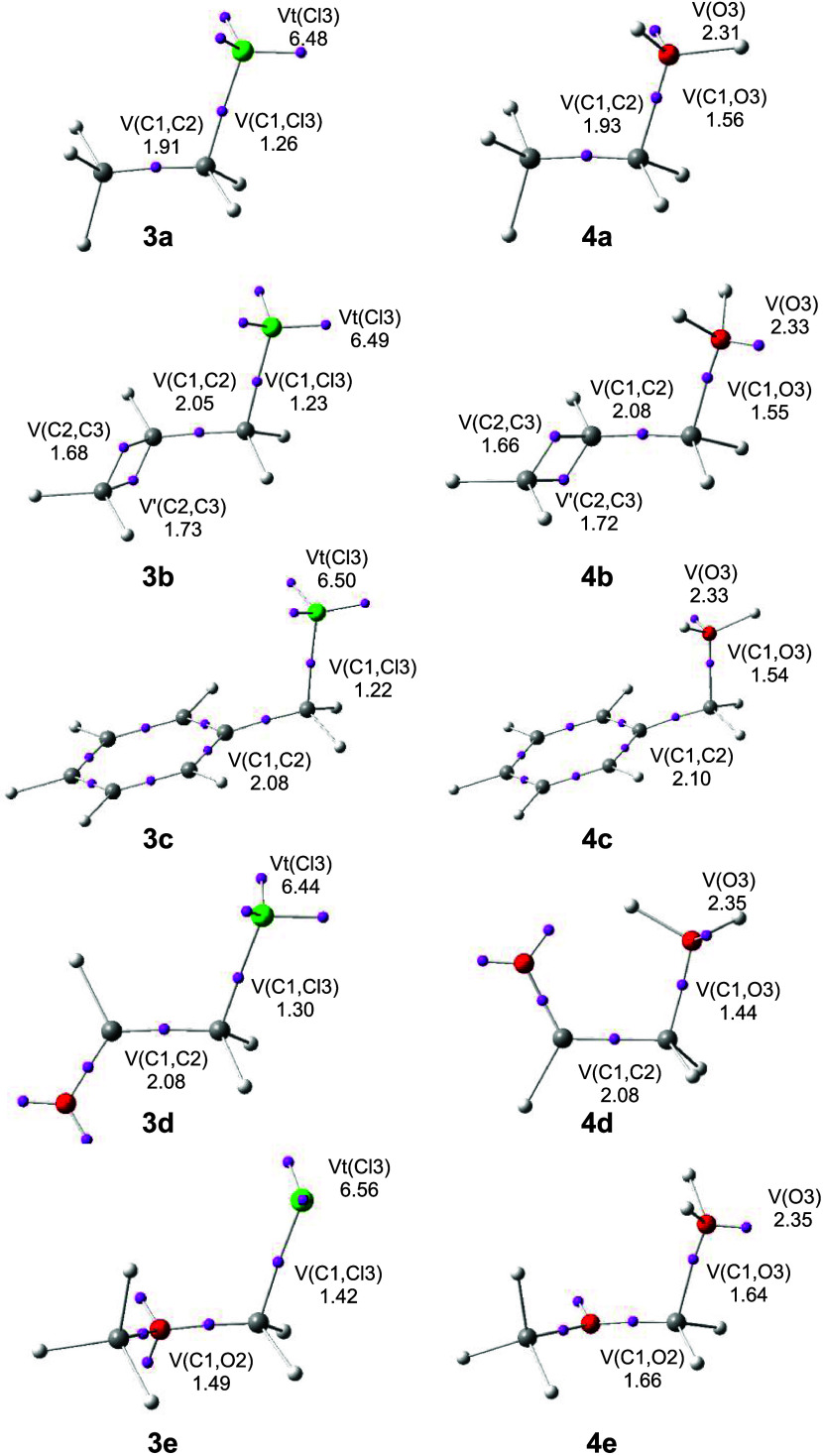
ωB97X-D/6-311+G­(d,p) ELF basin attractor positions, populations of the C–C and C–X (X = Cl or O) valence basins, and the total population of the V­(X; X = Cl or O) monosynaptic basins at the heteroatoms of DMCs **3a**–**e** and **4a**–**e**. Valence basin populations are given as the average number of electrons, e.

The more relevant valence basins characterizing DMCs **3a–e** are one V­(C1,C2) or V­(C1,O2) disynaptic basin, one V­(C1,Cl3) disynaptic basin, and two or three V­(Cl3) monosynaptic basins. For the series of chlorine derivatives **3a**–**e**, the populations of the V­(C1,C2) disynaptic basins range between 1.91 and 2.08 e, that of the V­(C1,Cl3) disynaptic basin range between 1.22 and 1.30 e, and the total population of the V­(Cl3) monosynaptic basins, that is, V_t_(Cl3), ranges between 6.44 and 6.50 e. These populations are somewhat different for chloromethoxymethane **3e**; they are 1.49 e (V­(C1,O2)), 1.42 e (V­(C1,Cl3)), and 6.56 e (V_t_(Cl3)). The populations of the V­(C1,Cl3) disynaptic and the V­(Cl3) monosynaptic basins indicate a strong polarization of electron density in the C1–Cl3 bonding region toward the electronegative Cl3 chlorine atom.

For the series of protonated alcohol derivatives **4a–d**, the population of the V­(C1,C2) disynaptic basin ranges between 1.93 and 2.10 e, that of the V­(C1,O3) disynaptic basins range between 1.44 and 1.56 e, and that of the V­(O3) monosynaptic basin ranges between 2.31 and 2.35 e. Again, these populations are somewhat different for protonated methoxymethanol **4e**; they are 1.66 (V­(C1,O2)), 1.64 (V­(C1,O3)), and 2.35 e (V­(O3)).

The ELF comparative analysis of the basin populations of DMCs **3a–e** and **4a–e** indicates that there are no significant changes in the electron density distribution in the C1–C2 and C1–X3 (X = Cl, O) bonding regions around the tetrahedral C1 carbon with the changes in the substituents R. Only in the methoxy derivatives **3e** and **4e** are a decrease of the electron density in the C1–O2 bonding region and an increase in the C1–O3 bonding region observed resulting from the polarization induced by the electronegative methoxy oxygen atom.

The ELF topological analysis of the electronic structure of DMCs **3a**–**e** and **4a**–**e** indicates that the substitution does not substantially modify the GS electron density distribution around the tetrahedral C1 carbon.^29^

### 2.2 Analysis of the Reactivity Indices at the GS of the Reagents

The analysis of the DFT-based reactivity indices^34,35^ at the GS of the reagents is a powerful tool for understanding the reactivity in polar reactions.^36^ The reactivity indices were calculated at the ωB97X-D/6-311+G­(d,p) computational level. A recent study demonstrated excellent linear correlations between the reactivity indices obtained at different levels of calculation and those obtained at the B3LYP/6-31G­(d) level.^37^ Due to the strong electrophilic and nucleophilic character of charged species, the reactivity indices were calculated in dimethyl sulfoxide (DMSO).^29,38^ The limits of the electrophilicity ω and nucleophilicity scales at the ωB97X-D/6-311+G­(d,p) computational level in DMSO are provided in Table S1. The global reactivity indices, namely, electronic chemical potential μ, chemical hardness η, global electrophilicity ω, and global nucleophilicity *N*, for DMCs **3a**–**e** and **4a**–**e** and the nucleophilic reagents are given in Table 1.

**Table 1:** ωB97X-D/6-311+G­(d,p) Electronic Chemical Potential μ, Chemical Hardness η, Electrophilicity ω, and Nucleophilicity *N* Indices, in eV, for DMCs **3a**–**e** and **4a**–**e** (R-CH_2_-LG) and the Nucleophilic Me_3_N: and Cl^–^ Species, Computed in DMSO

	R	LG	μ	η	ω	*N*
**4d**	CHO	H_2_O^+^	–5.94	11.29	1.56	–0.50
**4a**	CH_3_	H_2_O^+^	–5.87	13.99	1.23	–1.78
**4b**	CH_2_=CH	H_2_O^+^	–5.17	11.00	1.22	0.41
**4c**	Ph	H_2_O^+^	–4.78	9.77	1.17	1.41
**4e**	OCH_3_	H_2_O^+^	–5.38	12.84	1.12	–0.72
**3d**	CHO	Cl	–4.77	10.44	1.09	1.09
**3c**	Ph	Cl	–4.24	9.91	0.91	1.89
**3b**	CH_2_=CH	Cl	–4.35	10.91	0.87	1.27
**3e**	OCH_3_	Cl	–4.26	12.16	0.75	0.74
**3a**	CH_3_	Cl	–4.29	12.40	0.74	0.59
Me_3_N:			–3.18	10.04	0.50	2.88
Cl^–^			–3.11	11.58	0.42	2.18

The analysis of the electrophilicity ω indices of DMCs **3a**–**e** and **4a**–**e** indicates that while the protonated alcohols **4a**–**e**, as well as the chlorine methyl substituted compound **3d**, with ω ≥ 1.08 eV, are classified as strong electrophiles, the chlorine methyl substituted compounds **3b**–**e**, with ω < 1.08 eV, are classified as moderate electrophiles (see the limits of the electrophilicity ω scale in Table S1). As a result, these DMCs are expected not to have a tendency to react with nucleophiles. A similar behavior was found in methyl monosubstituted compounds.^29^

On the other hand, the analysis of the nucleophilicity *N* index of the two selected nucleophiles, 2.18 eV (Cl^–^) and 2.88 eV ((CH_3_)_3_N:), indicates that while the chloride anion Cl^–^, with *N* < 2.40 eV, is classified as a moderate nucleophile, the neutral trimethyl amine (CH_3_)_3_N:, with *N* ≥ 2.40 eV, is classified as a strong nucleophile (see the limits of the nucleophilicity *N* scale in Table S1).

Finally, the nucleofugality Λ indices of the H_2_O and Cl^–^ LGs computed at the ωB97X-D/6-311+G­(d,p) level in DMSO establish that the water H_2_O molecule (Λ = 1.41 eV) is markedly more effective LG than the chloride anion Cl^–^ (Λ = 0.77 eV) (see Table S1).

### 2.3 Analysis of the Reaction Paths Associated with the SN Reactions on DMCs **3a**–**e** and **4a**–**e**

To analyze the electronic effects of the substitution at the tetrahedral C1 carbon of DMCs **3a**–**e** and **4a**–**e**, three series of SN reactions were studied for each of the five substrates: (i) the symmetric SN reactions of the chlorine derivatives **3a**–**e** with chloride anion Cl^–^ as Nu, series I; (ii) the SN reactions of the chlorine derivatives **3a**–**e** with the good Nu trimethyl amine (CH_3_)_3_N:, series II; finally, (iii) the SN reactions of the protonated alcohols **4a**–**e** using water H_2_O molecule as an excellent LG with chloride anion Cl^–^, series III (see Scheme 5). Two reagents, one TS, and two products were located and characterized for each SN reaction. Consequently, all of these SN reactions take place through a one-step mechanism. Solvent effects play a crucial role in the kinetics of SN reactions involving charged species. To avoid the solvation of anionic nucleophiles, such as the chloride anion Cl^–^, nonprotic polar solvents such as DMSO are experimentally used.^4^ Therefore, all calculations were performed in DMSO.^29^ The ωB97X-D/6-311+G­(d,p) relative energies in DMSO are provided in Table 2, while the total energies are given in Table S3.

**Scheme 5:**
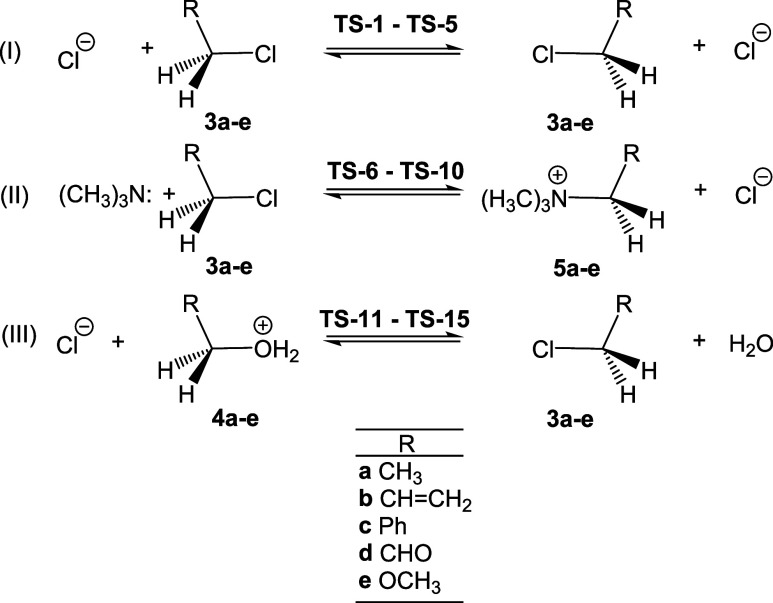
Three Series of SN Reactions Involving DMCs **3a**–**e** and **4a**–**e**

**Table 2:** ωB97X-D/6-311+G­(d,p) Relative Energies, in kcal·mol^–1^, in DMSO of the Stationary Points Involved in the SN Reactions of DMCs **3a**–**e** and **4a**–**e** ^a^

		R		CH_3_		CH=CH_2_		Ph		CHO		OCH_3_
Nu	LG		**n**	**3a**	**n**	**3b**	**n**	**3c**	**n**	**3d**	**n**	**3e**
		**R**		0.0		0.0		0.0		0.0		0.0
Cl^–^	Cl^–^	**TS-n**	**1**	23.5	**2**	19.9	**3**	18.7	**4**	18.3	**5**	10.7
		**P**		0.0		0.0		0.0		0.0		0.0
				**3a**		**3b**		**3c**		**3d**		**3e**
		**R**		0.0		0.0		0.0		0.0		0.0
(CH_3_)_3_N:	Cl^–^	**TS-n**	**6**	13.1	**7**	9.2	**8**	7.4	**9**	9.0	**10**	4.6
		**P**		–23.6		–23.5		–23.7		–19.2		–21.1
				**4a**		**4b**		**4c**		**4d**		**4e**
		**R**		0.0		0.0		0.0		0.0		0.0
Cl^–^	OH_2_	**TS-n**	**11**	7.5	**12**	3.6	**13**	2.2	**14**	6.7	**15**	–3.2
		**P**		–21.0		–22.4		–22.5		–22.4		–20.2

a See Scheme 5 for the nomenclature used in this table.

Along these SN reactions, a series of molecular complexes (MCs) can be found at the beginning and end of the reaction paths on the potential energy surfaces. Although these MCs have some significance under vacuum conditions, they are irrelevant in DMSO.^29^ However, in the SN reaction of protonated methoxymethanol **4e** with the chloride anion Cl^–^, the corresponding MC should be considered, as **TS-15** is located below the separated reagents (see Table 2).

The relative energies of the TSs for these SN reactions concerning the separated reagents range from 23.5 kcal·mol^–1^ (**3a** + Cl^–^) to −3.2 kcal·mol^–1^ (**4e** + Cl^–^), while the relative energies of the products lie in the narrow range of −19.2 kcal·mol^–1^ (**3d** + (CH_3_)_3_N:) to −23.7 kcal·mol^–1^ (**3c** + (CH_3_)_3_N:). Note that the symmetric SN reactions are isothermal. Some relevant conclusions can be obtained for the data given in Table 2: (i) the highest activation energy is associated with symmetric SN reaction **3a** + Cl^–^ due to the relatively low nucleophilic character and poor LG ability of the chloride anion Cl^–^; (ii) despite this, the SN reaction of the methoxy derivative **3e** with the chloride anion Cl^–^ is clearly favored, with Δ*E*_act_ = 10.7 kcal·mol^–1^; (iii) the activation energy for SN reaction of chloroethane **3a** with chloride anion Cl^–^ is 2.2 kcal·mol^–1^ higher in energy than that involving chloromethane **1a**,^24^ indicating that the introduction of a methyl CH_3_ group at the tetrahedral C1 carbon introduces a destabilizing factor; (iv) the activation energies for the series II of SN reactions involving the nucleophilic trimethyl amine (CH_3_)_3_N: are ca. 10 kcal·mol^–1^ lower than those for the series I of SN reactions involving chloride anion Cl^–^ as the nucleophile (see Table 2); (v) the electronic effects of substitution at the tetrahedral C1 carbon of the series II of SN reactions involving the nucleophilic trimethyl amine (CH_3_)_3_N: are similar to those in series I of SN reactions involving chloride anion Cl^–^ as the nucleophile. The activation energy for these SN reactions decreases in the order of CH_3_ > CH=CH_2_ ≈ CHO ≈ Ph > OCH_3_; (vi) series III of SN reactions, involving the departure of a water H_2_O molecule, are the most favored. The series III of SN reactions involving the departure of the water H_2_O molecule are ca. 15 kcal·mol^–1^ lower than those for series I, indicating the necessary use of an excellent LG. In fact, **TS-15**, associated with the SN reaction involving protonated methoxymethanol **4e**, is located below the separated reagents (see Table 2). However, if the formation of the corresponding MC is considered, Δ*E* = −3.4 kcal·mol^–1^, the activation energy becomes slightly positive at 0.3 kcal·mol^–1^; and finally (vii) in the series III of SN reactions the activation energies for DMC **4b** (R = CH=CH_2_, **TS-12** Δ*E*_act_ = 3.6 kcal·mol^–1^) and DMC **4c** (R = Ph, **TS-13** Δ*E*_act_ = 2.2 kcal·mol^–1^) are lower than that for DMC **4d** (R = CHO, **TS-14** Δ*E*_act_ = 6.9 kcal·mol^–1^), indicating that the CH=CH_2_ and Ph groups have a more stabilizing role than the CHO group in SN reactions involving an excellent LG.

The optimized geometries of the TSs involved in the nonsymmetric SN reactions are given in Figure 2, while those for the five symmetric TSs are shown in Figure S1. At the symmetric TSs, the C1–Cl distances vary from 2.32 Å at **TS-4** to 2.62 Å at **TS-5**. The large distance found in the most favorable **TS-5** indicates that the elimination of the chloride anion Cl^–^ takes place without the participation of the other chloride anion Cl^–^. These geometrical behaviors indicate that **TS-5** is associated with an S_N_1 mechanism. On the other hand, the C1–Cl distance in the second most favorable **TS-4** is the shortest. The geometries of the TSs involving DMCs **3a**–**c** and **4a**–**c** (R = (**a**) CH_3_, (**b**) CH=CH_2_, and (**c**) Ph) are quite similar in the two pairs of nonsymmetric reactions (see Figure 2). At the SN reactions involving nucleophilic trimethyl amine (CH_3_)_3_N:, the C1–Cl distances are between 2.30 and 2.32 Å, and the C1–N distances are between 2.22 and 2.27 Å. At the SN reactions involving the LG water H_2_O molecule, the C1–O3 distances are between 1.96 and 1.99 Å, and the C1–Cl distances are between 2.56 and 2.74 Å. The C1–N distances at the SN reactions with (CH_3_)_3_N:, series II, and the C1–Cl distances at the SN reactions with water H_2_O LG, series III, increase with TS stabilization. For the TSs involving the carbonyl derivatives **3d** (**TS-9**) and **4d** (**TS-14**) (R = CHO), these distances are shorter, while in TSs involving methoxy derivatives **3e** (**TS-10**) and **4e** (**TS-15**) (R = OH_2_), they are longer, except for the C1–O3 distance in **TS-15**, which is comparable to that in **TS-14**. The large C1–Cl distance at **TS-15**, 3.131 Å, indicates that this TS is mainly associated with the elimination of the water H_2_O molecule.

**Figure 2:**
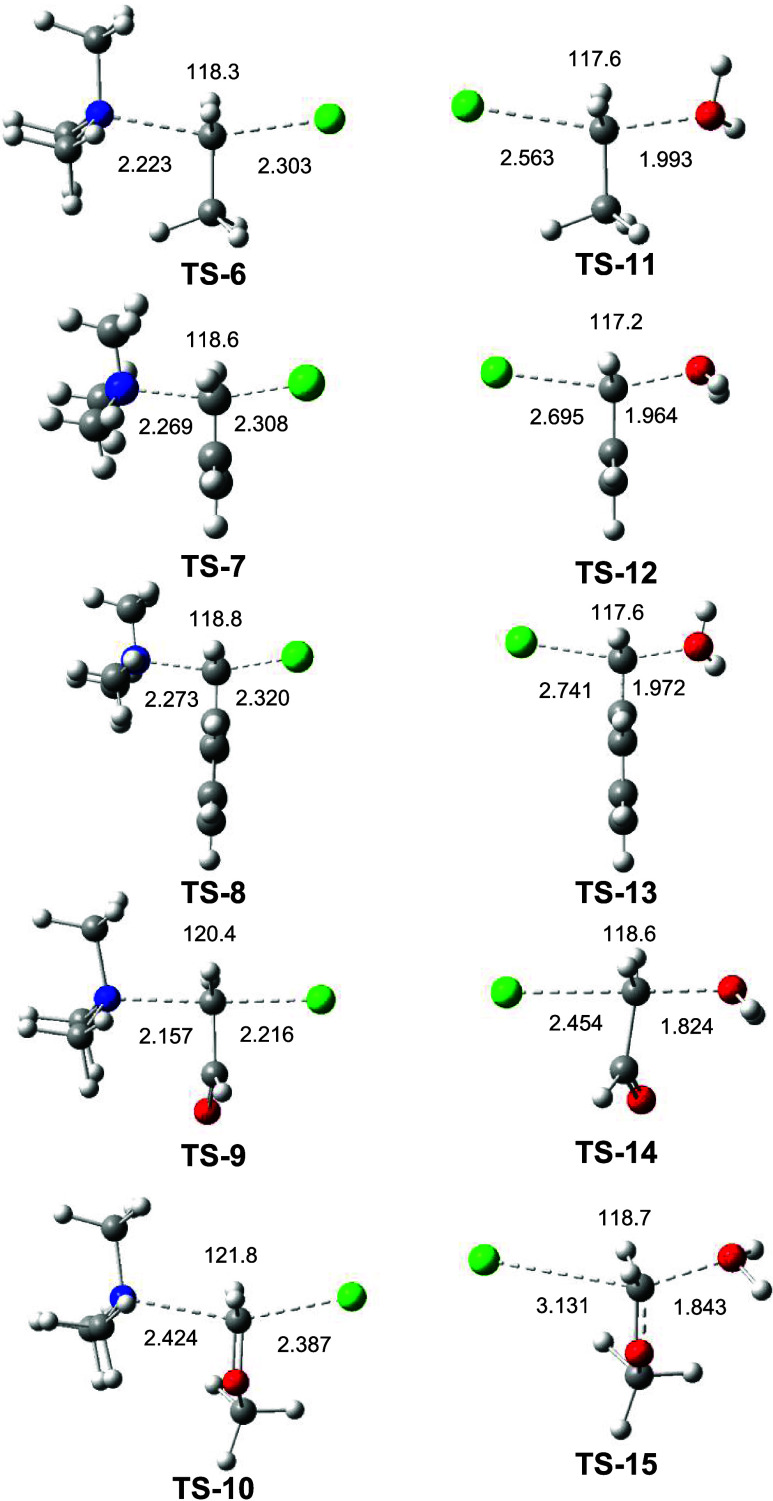
ωB97X-D/6–311+G­(d,p) optimized geometries of the nonsymmetric TSs. Distances are given in angstroms Å, while H–C–H bond angles are given in degrees.

In general, the C1–Cl distances at the TSs of series III, which involve the departure of the water H_2_O molecule, are larger than the C1–N distances at the TSs of series II, which involve the departure of the chlorine anion Cl^−^. This suggests that the presence of water H_2_O LG diminishes the participation of nucleophilic species. These behaviors indicate a shift in the molecular mechanism from S_N_2 to S_N_1 with the participation of an excellent LG.

At all 15 TSs, the RCH_2_ framework exhibits a nearly trigonal planar structure, corresponding to that of a carbocation species (see the H–C–H angle near 120° at the TSs in Figures 2 and S1).^29^

Next, the global electron density transfer^39^ (GEDT) taking place from the Nu and the LG toward the central substituted tetrahedral C1 carbon at the TSs of these SN reactions was evaluated. Recently, it has been shown that the GEDT plays a decisive role in polar organic reactions.^40^ The sums of the natural population analysis (NPA) charges for the atoms belonging to Nu, LG species, and the RCH_2_ framework are provided in Table 3.

**Table 3:** ωB97X-D/6-311+G­(d,p) NPA Total Atomic Charges, as the Average Number of Electrons, e, of the Nu, LG, and RCH_2_ Frameworks at the 15 TSs

	R	Nu	RCH_2_	LG
**TS-1**	CH_3_	–0.71	0.42	–0.71
**TS-2**	CH=CH_2_	–0.72	0.44	–0.72
**TS-3**	Ph	–0.72	0.45	–0.72
**TS-4**	CHO	–0.58	0.16	–0.58
**TS-5**	OCH_3_	–0.84	0.67	–0.84
**TS-6**	CH_3_	0.23	0.38	–0.61
**TS-7**	CH=CH_2_	0.22	0.37	–0.60
**TS-8**	Ph	0.23	0.38	–0.61
**TS-9**	CHO	0.28	0.20	–0.48
**TS-10**	OCH_3_	0.12	0.58	–0.70
**TS-11**	CH_3_	–0.78	0.56	0.22
**TS-12**	CH=CH_2_	–0.84	0.60	0.24
**TS-13**	Ph	–0.86	0.62	0.27
**TS-14**	CHO	–0.65	0.34	0.31
**TS-15**	OCH_3_	–0.97	0.70	0.27

At the symmetric TSs belonging to the series I of SN reactions, the total charge of the RCH_2_ framework is 0.42 e (**TS-1**), 0.44 e (**TS-2**), 0.45 e (**TS-3**), 0.16 e (**TS-4**), and 0.67 e (**TS-5**). The deviation from the charge of 1.00 e, corresponding to a central carbocation RCH_2_^+^ framework, arises from the GEDT occurring from the two neighboring nucleophilic chloride anions Cl^–^, which carries a charge of less than −1.00 e. The changes in the total charge of the RCH_2_ framework at the five symmetric TSs are analyzed. The RCH_2_ frameworks in **TS-1**, **TS-2**, and **TS-3** show similar total charges that increase slightly in that order. This increase in total charge at these SN reactions is accompanied by a stabilization of the corresponding TS (Table 2), which becomes earlier (Figure S1). **TS-4** and **TS-5** show different behaviors; while **TS-4** presents the highest GEDT value, with a total charge of 0.16 e at the HCOCH_2_ framework, **TS-5** presents the lowest GEDT value, with a total charge of 0.67 e at the CH_3_OCH_2_ framework. Note that the CH_3_O group is the most stabilizing one. These GEDT values indicate that the stabilizing factor of the carbonyl CHO and methoxy CH_3_O groups differs from those of the others. Thus, the C1–O2 distance at **TS-5**, 1.270 Å, is markedly shorter than that in the methoxy derivative **4e**, 1.358 Å, indicating a strong interaction between the central C1 and the methoxy O2 atoms at this TS. This finding explains why, in the three series of SN reactions, the TSs involving the methoxide derivatives **3e** and **4e** present lower relative energies (see Table 2).

The analysis of the GEDT to the RCH_2_ frameworks of the nonsymmetric TSs associated with the series II and III of SN reactions reveals a similar trend to that observed in the series I, indicating that the substitution in the tetrahedral C1 carbon causes similar electronic effects at the corresponding TSs, independent of the nature of the Nu or the LG. Thus, as shown in Figure S2, a strong correlation exists between the NPA total atomic charge of the RCH_2_ frameworks at the TSs in the nonsymmetric series I of SN reactions and those in the symmetric ones: *R* ^2^ = 0.93 (series II) and 0.98 (series III).

Finally, the analysis of the GEDT to the RCH_2_ frameworks of the TSs associated with series I and III of SN reactions reveals that the TSs belonging to series III have more carbocation character. This behavior, together with the increases of C1–Cl distances in series III, points out the shifts in the molecular mechanism from an S_N_2 to an S_N_1 one.

### 2.4 ELF Topological Comparative Analyses of the TSs Associated with the SN Reactions on DMCs **3a–e** and **4a–e**

In this section, an ELF topological analysis of the electronic structure of the 15 TSs was performed to characterize the evolution of the rupture of the C–X single bond and the formation of the C–Y single bond at these TSs. Figure 3 shows the ELF basin attractor positions, together with the most relevant valence basin populations, of the 10 nonsymmetric TSs, while those of the five symmetric TSs are given in Figure S3.

**Figure 3:**
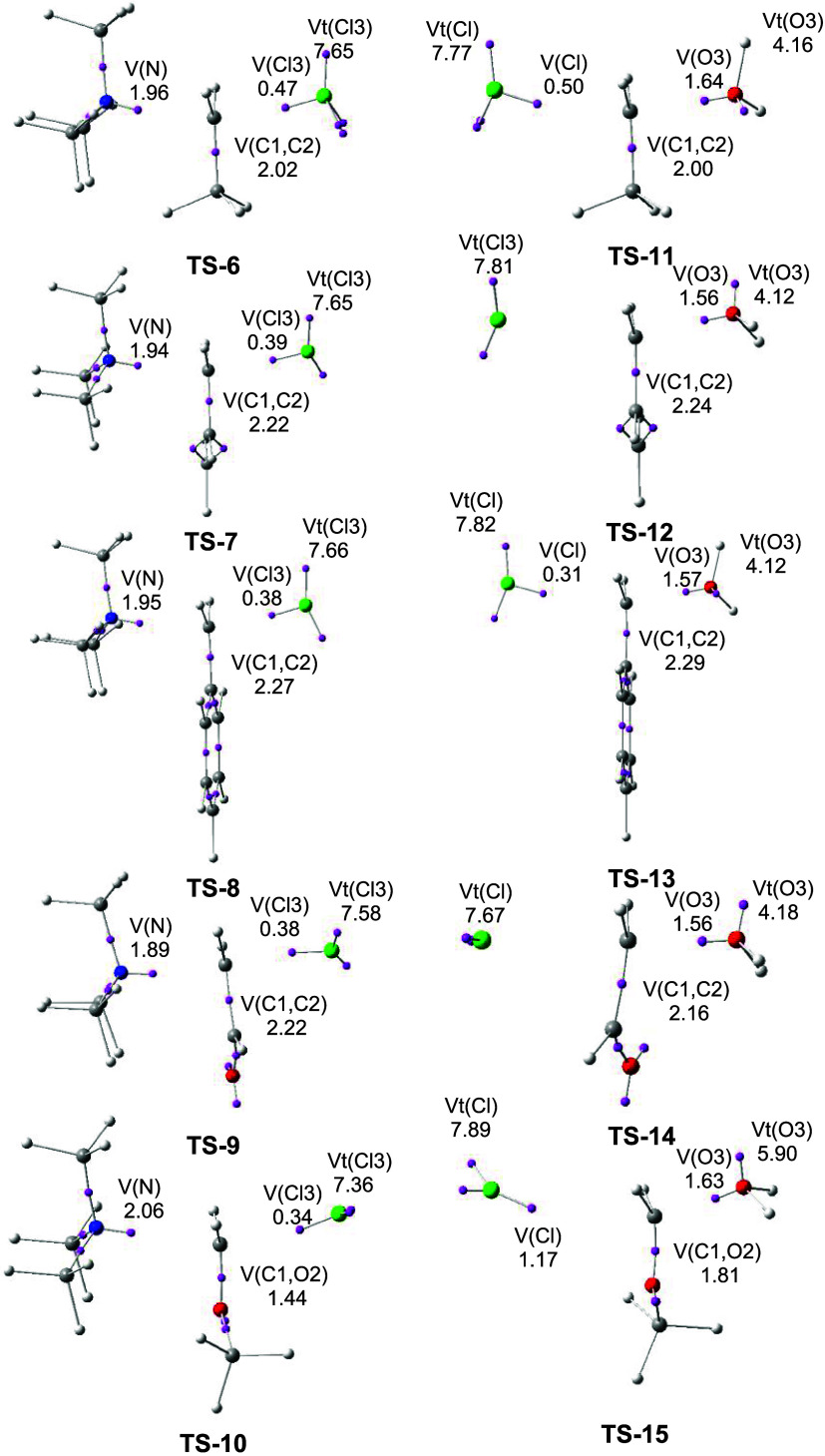
ωB97X-D/6-311+G­(d,p) ELF basin attractor positions, populations of the C–X valence basins, and the total population of the V­(X) monosynaptic basins at the heteroatoms of the TSs associated with series II and III of nonsymmetric SN reactions. Valence basin populations are given as the average number of electrons, e.

The ELF analysis of the 15 TSs shows the presence of one V­(Cl) or one V­(N) monosynaptic basin in the C1–Cl or C1–N interacting regions involving the chloride anion Cl^–^ and trimethyl amine (CH_3_)_3_N: as the Nu and one V­(Cl3) or one V­(O3) monosynaptic basin in the C1–Cl3 or C1–O3 interacting bonding regions involving chloride anion Cl^–^ or water H_2_O molecule as the LG, respectively. In addition, the chloride anions Cl^–^ can present one or two additional V­(Cl) monosynaptic basins. In **TS-5** and **TS-14**, however, the chloride atom does not present any V­(Cl) monosynaptic basin directed toward the C1 carbon. No V­(C1,Z) (Z = Cl, N, O) disynaptic basins are observed in any of the 15 TS, indicating that while the C–Y (Y = Cl, O) single bond involving the LG has been broken, the formation of the new X–C (X = Cl, N) single bond involving the Nu has not yet begun. A similar behavior was found in the SN reactions of the methyl monosubstituted compounds **1a**–**d**.^29^

At the symmetric TSs of series I of SN reactions, the population of the V­(Cl) monosynaptic basins ranges from 0.23 to 0.37 e. In the nonsymmetric TSs of the series II of SN reactions, the population of the V­(N) monosynaptic basins, which are associated with the nitrogen lone pair of the trimethyl amine (CH_3_)_3_N:, is found in the range of 1.89–2.06 e, while at TSs of the series III of SN reactions, the population of the V­(O3) monosynaptic basins, which are associated with one of the two oxygen lone pairs of the water H_2_O molecule, ranges from 1.56 to 1.64 e. Consequently, except for the most favorable **TS-15**, the other 14 TSs show a very similar electronic structure.

The V­(C1,C2) disynaptic basins at the TSs of three studied series of SN reactions of DMCs **3a**–**e** and **4a**–**e** show a similar population, indicating that the CH_3_, CH=CH_2_, Ph, and CHO groups exert similar electronic effects in the three series of SN reactions. Only the V­(C1,O2) disynaptic basins at **TS-5**, **TS-10**, and **TS-15** having the methoxy OCH_3_ group show different populations of 2.07, 1.44, and 1.81 e, respectively, indicating different participation of the methoxy oxygen atom in the stabilization of the central C1 carbon atom in these TSs.

Finally, a comparison of the populations of the V­(C1,C2) disynaptic basins at the symmetric **TS-2**, **TS-3**, and **TS-4** (see Figure S1) with those of DMCs **3b**, **3c**, and **3d** at the GS (see Figure S1) reveals an increase of ca. 0.2 e in the TS populations, indicating some electron delocalization between the central C1 carbon and the CH=CH_2_, Ph, and CHO substituents. The larger deviation at the most favorable **TS-5**, ca. 0.6 e, reflects a strong electronic interaction between the C1 carbon and the methoxy O2 oxygen atom.

### 2.5 RIAE Analysis of the Symmetric SN Reactions of the Chloromethyl-Substituted Compounds **3a**–**e** with Chlorine Anion Cl^–^

Finally, to determine the electronic effects of the substituents in these SN reactions, an RIAE analysis^26^ of the five TSs of the symmetric series I of SN reactions of the chloromethyl-substituted compounds **3a**–**e** with chloride anion Cl^–^ was performed. The symmetric SN reaction of chloromethane **1a** (R = H) with chloride anions Cl^–^ via **TS-16** was also studied as a reference for these SN reactions.^29^ The RIAE analysis was carried out at the M06-2X/6-311+G­(d,p) computational level, as this functional is required by the IQA^32^ calculations. A plot of the computed M06-2X/6-311+G­(d,p) activation energies in DMSO versus those computed at ωB97X-D/6-311+G­(d,p) in DMSO shows a good linear correlation with an *R* ^2^ = 0.96 (see Figure S4 and Table S4). Although the M06-2X activation energies are between 2.4 and 5.2 kcal·mol^–1^ higher than those of the ωB97X-D ones, this comparative analysis indicates that the substituent effects on the activation energies in series I of SN reactions are similarly assessed by both functionals. The gas-phase M06-2X/6-311+G­(d,p) ξ*E*_intra_^X^ intra-atomic, ξ*E*_inter_^X^ interatomic, and ξ*E*_total_^X^ total energies of the RCH_2_Cl and chlorine frameworks of the six TSs, relative to the corresponding MCs, are given in Table 4. The theoretical background to the RIAE analysis can be found in the Supporting Information.

**Table 4:** M06-2X/6-311+G­(d,p) Gas-Phase ξ*E*_intra_^X^ Intra-Atomic, ξ*E*_inter_^X^ Interatomic, and ξ*E*_total_^X^ Total Energies, in kcal·mol^–1^, of the RCH_2_Cl and the Nucleophilic Chlorine Frameworks at the TSs Relative to the MCs^a^

R	*f*(X)	ξ*E*_intra_^X^	ξ*E*_inter_^*X*^	ξ*E*_total_^*X*^	ξ*E*_total_^RCH_2_Cl+Cl^
**TS-16** H	RCH_2_Cl	–32.1	45.0	12.9	13.5
	Cl	26.2	–25.6	0.6	
**TS-1** CH_3_	RCH_2_Cl	–37.2	52.9	15.7	17.2
	Cl	26.9	–25.4	1.5	
**TS-2** CH=CH_2_	RCH_2_Cl	–40.7	50.2	9.5	14.4
	Cl	27.4	–22.5	4.9	
**TS-3** Ph	RCH_2_Cl	–37.5	46.1	8.6	12.4
	Cl	28.5	–24.7	3.8	
**TS-4** CHO	RCH_2_Cl	–28.2	34.2	6.0	12.4
	Cl	27.2	–20.8	6.4	
**TS-5** OCH_3_	RCH_2_Cl	47.6	–37.4	10.2	8.4
	Cl	16.3	–18.2	–1.9	

a The sum of the ξ*E*_total_^X^ energies of both frameworks, that is, ξ*E*_total_^RCH_2_Cl+Cl^, yields the RIAE activation energies.

RIAE analysis of **TS-16**, associated with the SN reaction of chloromethane **1a** with chloride anion Cl^–^, used as the energy reference for the study of substituent effects, shows that the ξ*E*_total_^RCH_2_Cl^ total energies of the RCH_2_Cl framework, 12.9 kcal·mol^–1^, are the main factor responsible of the activation energy of this SN reaction (see Table 4). Despite the strong intra-atomic stabilization of the RCH_2_Cl framework, ξ*E*_intra_^RCH_2_Cl^ = −32.1 kcal·mol^–1^, the higher interatomic destabilization of this framework is responsible for the activation energy, ξ*E*_inter_^RCH_2_Cl^ = 45.0 kcal·mol^–1^. A detailed analysis of the interatomic energies responsible for the ξ*E*_inter_^RCH_2_Cl^ energies indicates that the interatomic destabilization of the C1 carbon, ξ*E*_inter_^C1^ = 17.0 kcal·mol^–1^, and that of the chloride anion Cl^–^ LG, ξ*E*_inter_^Cl^ = 34.8 kcal·mol^–1^, are the primary contributors to the RIAE activation energy.

The inclusion of a methyl CH_3_ group in the tetrahedral C1 carbon of chloromethane **1a** increases the RIAE activation energy of the SN reaction of chloroethane **3a** by 3.7 kcal·mol^–1^. RIAE analysis of **TS-1** shows that the electronic factors responsible for the RIAE activation energy of **TS-16** are also present in the former (see Table 4). Although the intra-atomic interactions at the CH_3_CH_2_Cl framework are more stabilizing, ξ*E*_intra_^RCH_2_Cl^ = −37.2 kcal·mol^–1^, the interatomic ones are more destabilizing, ξ*E*_inter_^RCH_2_Cl^ = 52.9 kcal·mol^–1^. As a result, the ξ*E*_total_^RCH_2_Cl^ total energies increase by 2.8 kcal·mol^–1^ with the inclusion of the methyl CH_3_ group. A detailed analysis of the interatomic energies associated with the atoms belonging to the CH_3_CH_2_Cl framework of **TS-1** indicates that the destabilization of the C1 carbon by 19.3 kcal·mol^–1^ is the main factor responsible for the increase in the activation energy. On the other hand, an analysis of the interatomic energies between the nucleophilic Cl framework and the nearest hydrogen atom of the methyl substituent of the CH_3_CH_2_Cl framework of **TS-1** shows a weak Cl–H stabilizing interaction of 2.1 kcal·mol^–1^, indicating the absence of any significant repulsive steric interaction during the approach of the chloride anion Cl^–^ to chloroethane **3a**.

The inclusion of a vinyl CH = CH_2_ group in the tetrahedral C1 carbon of chloromethane **1a** increases the RIAE activation energy of the SN reaction of 3-chloropropene **3b** by 1.0 kcal·mol^–1^. Although the ξ*E*_total_^RCH_2_Cl^ total energies of the CH_2_=CHCH_2_Cl framework at **TS-2** are 3.4 kcal·mol^–1^ more stabilizing than those at **TS-16**, the ξ*E*_total_^Cl^ total energies of the nucleophilic Cl framework are 4.3 kcal·mol^–1^ more destabilizing. On the other hand, the inclusion of a phenyl Ph group in the tetrahedral C1 carbon of chloromethane **1a** decreases the RIAE activation energy of the SN reaction of benzyl chloride **3c** by 1.1 kcal·mol^–1^. The substitution of the vinyl CH=CH_2_ group in 3-chloropropene **3b** by the phenyl Ph one in benzyl chloride **3c** reduces the ξ*E*_total_^X^ total energies of both frameworks. As a result, the SN reaction of benzyl chloride **3c** with chloride anions Cl^–^ becomes more favorable.

The inclusion of the carbonyl CHO group in the tetrahedral C1 carbon of chloromethane **1a** causes a similar decrease of the RIAE activation energy in the SN reaction of 2-chloroethanal **3d** to that caused by the phenyl Ph group present in benzyl chloride **3c** but through a different molecular mechanism. While the ξ*E*_total_^Cl^ total energies of the nucleophilic Cl framework at **TS-4** are destabilized by 2.6 kcal·mol^–1^ compared to those at **TS-3**, the ξ*E*_total_^RCH_2_Cl^ total energies are stabilized by 2.6 kcal·mol^–1^. The NPA analysis given in Table 3 indicates that the total atomic charge at the CHOCH_2_ framework of **TS-4**, 0.16 e, is significantly lower than that at the PhCH_2_ framework in **TS-3**, 0.45e, indicating a higher GEDT at the former. Note that the CHOCH_2_ framework at **TS-4**, **TS-9**, and **TS-14**, involving the presence of the carbonyl CHO group, presents the highest GEDT among the 15 TSs (see Table 3). As commented on, the GEDT taking place at the TSs is the main factor responsible for the reduction of activation energies in polar organic reactions.^40^ Consequently, although the carbonyl HCO and phenyl Ph groups generally have different electronic effects, they result in the same RIAE activation energies for these symmetric SN reactions. Note, however, that in series II and III of nonsymmetric SN reactions, the stabilizing effect of the phenyl Ph group is greater than that of the carbonyl CHO group (see Table 2).

An interesting case is the SN reaction of chloromethoxymethane **3e**, which experiences a reduction of the RIAE activation energy of 5.1 kcal·mol^–1^ with respect to that of chloromethane **1a**. While the ξ*E*_total_^RCH_2_Cl^ total energies of the CH_3_OCH_2_Cl framework in **TS-5** have been reduced to 10.2 kcal·mol^–1^, the ξ*E*_total_^Cl^ total energies of the chloride anion Cl^–^ framework become stabilizing by –1.9 kcal·mol^–1^. Two behaviors need to be commented on. While the ξ*E*_inter_^RCH_2_Cl^ interatomic energies of the CH_3_OCH_2_Cl framework in **TS-5** become negative by 37.4 kcal·mol^–1^, the unfavorable ξ*E*_intra_^Cl^ intra-atomic energies of the nucleophilic chloride anion Cl^–^ framework experience a reduction of ca. 10 kcal·mol^–1^. Despite the ξ*E*_intra_^RCH_2_Cl^ intra-atomic energies becoming highly unfavorable by 47.6 kcal·mol^–1^, the highly favorable interatomic energies of the CH_3_OCH_2_Cl framework, ξ*E*_inter_^RCH_2_Cl^ = −37.4 kcal·mol^–1^, together with the less unfavorable ξ*E*_intra_^Cl^ intra-atomic energies are responsible for the favorable substituent effect of the methoxy OCH_3_ group in these SN reactions (see Table 4). A detailed analysis of the factors contributing to the negative ξ*E*_inter_^RCH_2_Cl^ interatomic energies at **TS-5** indicates that the ξ*E*_inter_^C1^ interatomic energy of the C1 carbon, −33.8 kcal·mol^–1^, and the ξ*E*_inter_^O2^ interatomic energy of the methoxy oxygen, −50.1 kcal·mol^–1^, are the main factors responsible for this C1–O2 interatomic stabilization. This finding suggests that the electrostatic interactions between the negatively charged methoxy O2 oxygen and the positively charged C1 carbon at **TS-5** are the main factors responsible for the strong activation effect of the methoxy OCH_3_ group. This stabilizing interaction causes the TSs involving the methoxy derivatives **3e** and **4e** to present the lowest GEDT and the highest acceleration of these SN reactions (see Tables 2 and 3).

Finally, Figure 4 shows a graphical representation of the ξ*E*_total_^X^ total energies of the RCH_2_Cl and Cl frameworks at six TSs, as shown in blue and red, respectively. The ξ*E*_total_^RCH_2_Cl+Cl^ total energies, shown in black, correspond to the RIAE activation energies of these SN reactions. Except for the reaction involving 2-chloroethanal **3d**, **TS-4**, in the other five reactions, the unfavorable ξ*E*_total_^RCH_2_Cl^ total energies of the RCH_2_Cl frameworks dominate the RIAE activation energies. For the SN reactions of 3-chloropropene **3b**, **TS-2**, and benzyl chloride **3c**, **TS-3**, although the ξ*E*_total_^RCH_2_Cl^ total energies decrease with respect to the SN reaction of chloromethane **1a**, **TS-16**, the ξ*E*_total_^Cl^ total energies increase. As can be seen in the comparison between the SN reactions of benzyl chloride **3c**, **TS-3**, and 2-chloroethanal **3d**, **TS-4**, which have similar RIAE activation energies, while the ξ*E*_total_^RCH_2_Cl^ total energies decrease in **TS-4**, the ξ*E*_total_^Cl^ total energies become more unfavorable. Interestingly, in the most favorable SN reaction involving chloromethoxymethane **3e**, **TS-5**, while the ξ*E*_total_^RCH_2_Cl^ total energies present the highest energy among the CH=CH_2_, CHO, and Ph groups, the ξ*E*_total_^Cl^ total energies become negative and, consequently, favorable. This is a consequence of the reduced participation of the nucleophilic Cl framework at symmetric **TS-5**. Note that both C1–Cl distances, 2.620 Å, along with the ELF topological analysis of this TS, suggest that this SN reaction takes place via an S_N_1 mechanism (see **TS-5** in Figures S1 and S3).

**Figure 4:**
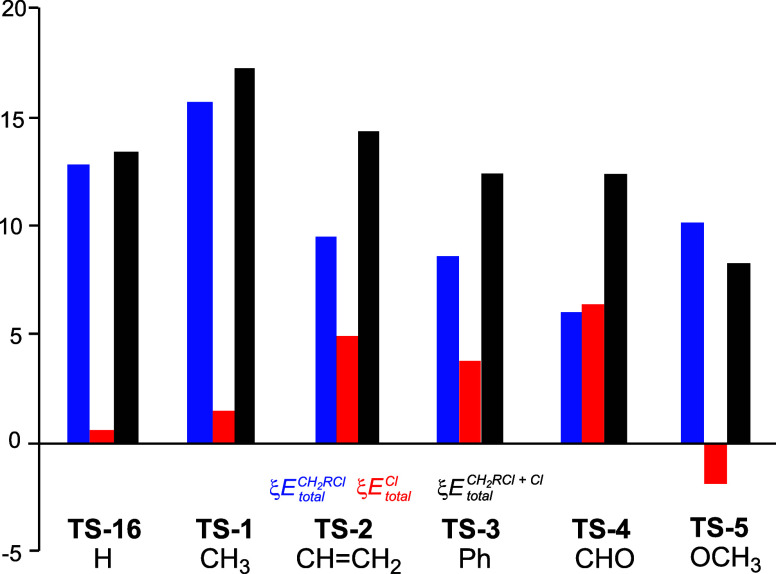
Graphical representation of the sets of the ξ*E*_total_^RCH_2_Cl^, ξ*E*_total_^Cl^, and ξ*E*_total_^RCH_2_Cl+Cl^ total energies in that order for the TSs associated with the SN reactions of chloromethane **1a** and DMCs **3a–e** with chloride anion Cl^–^. The ξ*E*_total_^RCH_2_Cl+Cl^ energies correspond to the RIAE activation energies of these SN reactions. ξ*E*_total_^X^ energies of the RCH_2_Cl and Cl frameworks are colored blue and red, respectively. The black bar represents the ξ*E*_total_^RCH_2_Cl+Cl^ energies. ξ*E*_total_^X^ energies are given in kcal·mol^–1^.

## 3 Conclusions

The energetic and electronic effects of the CH_3_, CH=CH_2_, Ph, CHO, and OCH_3_ groups attached to the tetrahedral carbon involved in the SN reactions of five monosubstituted chloromethanes **3a–e** and five protonated monosubstituted methanol derivatives **4a–e** have been studied within MEDT at the ωB97X-D/6-311+G­(d,p) computational level. An ELF topological analysis of DMCs **3a–e** and **4a–e** indicates that there is no significant change in the electron density distribution in the C1–C2 and C1–X3 (X = Cl or O) bonding regions around the tetrahedral C1 carbon with the change of the substituent. Analysis of the DFT-based reactivity indices indicates that while DMCs **4a–e** are classified as strong electrophiles due to their cationic nature, neutral DMCs **3a–e**, with ω **<** 1.08 eV, are classified as moderate electrophiles, suggesting that they have no tendency to react with nucleophiles.

Analysis of the activation energies of the three series of SN reactions, ranging from 0.3 to 23.5 kcal·mol^–1^, shows similar electronic effects of the substituents attached to the tetrahedral C1 carbon. The activation energies for these SN reactions decrease in the order CH_3_ > H > CH=CH_2_ ≈ CHO > Ph > OCH_3_. While the CH=CH_2_, Ph, and CHO groups exhibit a similar activating effect in series I and II of the SN reactions involving the chloride anion Cl^–^ as the LG, the OCH_3_ group exhibits a highly effective activating effect in all three series. The use of the nucleophilic trimethyl amine (CH_3_)_3_N: in series II of SN reactions reduces the activation energies by 10 kcal·mol^–1^ compared to those using the chloride anion Cl^–^ as the Nu in series I, whereas the use of the highly effective LG, the water H_2_O molecule, reduces the activation energies of series III of SN reactions by more than 15 kcal·mol^–1^. These energy results suggest that although a good Nu is required in these SN reactions, the presence of a very good LG is essential to favor its early departure, thereby electrophilically activating the saturated tetrahedral C1 carbon atom for nucleophilic attack.

While the geometries of the TSs of the SN reactions involving the CH_3_, CH=CH_2_, and Ph groups are very similar, those involving the CHO and OCH_3_ groups are somewhat different. The RCH_2_ framework at the 15 TSs exhibits a nearly trigonal planar structure, corresponding to that of a CH_2_R^+^ carbocation species.^29^ All TSs present a total charge at the RCH_2_ framework of less than 0.70 e, as a consequence of an electron density donation from the Nu and LG species. The GEDT taking place at the TSs varies depending on the stabilizing effect of the substituents.

A comparative ELF topological analysis of the electron density of the 15 TSs indicates that, except for the most favorable **TS-15**, the other TSs present a similar electronic structure. Interestingly, both geometrical and ELF topological analyses of **TS-15** suggest that it is associated with an S_N_1 mechanism; however, all attempts to locate the corresponding carbocation intermediate were unsuccessful. This finding supports the close relationship between the S_N_1 and S_N_2 mechanisms recently found in the SN reactions of the methyl derivatives **1a,c** shown in Scheme 3.^29^

Finally, RIAE analysis of symmetric series I of SN reactions of the chloromethyl-substituted compounds **3a**–**e** with chloride anion Cl^–^ allows the establishment of the electronic effects of the five selected substituents on reducing the activation energies. Although the carbonyl CHO and phenyl Ph groups cause the same reduction in the RIAE activation energies, their electronic effects are quite different at the corresponding TSs. The strong GEDT occurring at **TS-4**, which contains the CHO group, is responsible for the decrease in its activation energy. On the other hand, the strong stabilization of the ξ*E*_*inter*_^*x*^ interatomic energies of the C1 carbon and O3 oxygen atoms of the CH_3_OCH_2_Cl framework, together with the reduction of the unfavorable ξ*E*_intra_^Cl^ intra-atomic energies of the nucleophilic Cl^–^ at **TS-5**, explains the strong activating effect of the methoxy OCH_3_ group in these SN reactions.

The present MEDT study emphasizes that both the electronic effects of the substituents at the primary tetrahedral carbons and the use of an excellent LG can shift the molecular mechanism of SN reactions of primary substituted carbons from an S_N_2 to an S_N_1 one. Since the tetrahedral carbon undergoing the SN reaction is cationic in nature at the TS, the involvement of the attacking nucleophile in its stabilization determines the molecular mechanism.

## 4 Computational Details

This MEDT study has been carried out by performing quantum chemical calculations within density functional theory (DFT).^41^ The ωB97X-D^42^ functionals, together with the standard 6-311+G­(d,p)^43^ basis set, which includes and diffuses d-type polarization for second-row elements and p-type polarization functions for hydrogens, were used throughout this MEDT study. In the RIAE analysis,^26^ the stationary points involved in the five selected symmetric SN reactions were also computed using the M06-2X functional.^44^ The TSs were characterized by the presence of only one imaginary frequency. The Berny method was used in optimizations.^45,46^ The intrinsic reaction coordinate^47^ (IRC) calculations were performed to establish the unique connection given between the TSs and the corresponding minima.^48,49^ Solvent effects were considered by full optimization of the gas phase structures at the same computational level using the polarizable continuum model (PCM)^50,51^ in the framework of the self-consistent reaction field (SCRF)^52–54^ and DMSO as solvent.

The GEDT^39^ values were computed using the equation GEDT­(*f*) = ∑q_f_, where q are the natural charges^55,56^ of the atoms belonging to one of the two frameworks (f) at the TS geometries. Global and local CDFT indices^34–36^ were calculated using the equations in ref 35.

The Gaussian 16 suite of programs was used to perform the calculations.^57^ ELF^25^ analyses of the ωB97X-D/6-311+G­(d,p) monodeterminantal wave functions were done by using the TopMod5 ^58^ package with a cubical grid of step size of 0.1 Bohr. Molecular geometries and ELF basin attractors were visualized by using GaussView program.^59^

The IQA^32^ energy decomposition analysis used in the RIAE analysis was performed with the AIMAll package^60^ using the corresponding M06-2X/6-311+G­(d,p) monodeterminantal pseudowave functions. Note that the AIMAll package allows the use of only the B3LYP and the M06-2X functionals.

## Supplementary Material



## Data Availability

The data underlying this study are available in the published article and its Supporting Information.

## Notes

Theoretical background of the relative interacting atomic energy (RIAE) analysis. Figure with the ωB97X-D/6-311+G­(d,p) optimized geometries of the symmetric TSs. Plots of the NPA total atomic charge of the RCH_2_^+^ frameworks of the TSs of the series II and III of SN reactions with respect to those of the series I. Figure with the ωB97X-D/6-311+G­(d,p) ELF basin attractor positions, populations of the C–X valence basins, and the sum of the population of the V­(X) monosynaptic basins at the heteroatoms of the TSs associated with the symmetric series I of SN reactions. Figure with the plot of the M06-2X/6-311+G­(d,p) activation energies in DMSO of the symmetric series I of SN reactions of DMCs **3a**–**e** with respect to the computed ωB97X-D/6-311+G­(d,p) activation energies in DMSO. Linear regression equations and Pearson correlation coefficient *R* ^2^ values from the plots of electrophilicity ω and nucleophilicity *N* indices of selected neutral molecules given in ref 35 computed at the ωB97X-D/6-311+G­(d,p) computational levels in DMSO vs those obtained at the B3LYP/6-31G­(d) level in vacuo, and the Lower, upper, and superior (super) limits of the electrophilicity and nucleophilicity ωB97X-D/6-311+G­(d,p) scales in DMSO. Table with the ωB97X-D/6-311+G­(d,p) total electronic energies of the stationary points involved in the SN reactions of DMCs **1a–e** and **2a–e**. ωB97X-D/6-311+G­(d,p) computed total energies in DMSO, imaginary frequencies, and Cartesian coordinates of the TS involved in SN reactions of DMCs **1a–e** and **2a–e**. (PDF)
