# Supplementary material for: Advanced Molecular Electron Density Theory Study of the Substituent Effects in Nucleophilic Substitution Reactions
Source: ACS Omega. 2025 Jul 7;10(28):30194–206. doi: 10.1021/acsomega.5c00957 (PMC12290940; doi:10.1021/acsomega.5c00957)
Supplement: Supplementary file 1 [file ao5c00957_si_001.pdf]

Supporting Information

Advanced Molecular Electron Density Theory Study of the Substituent Effects in Nucleophilic Substitution Reactions

Luis R. Domingo,[1,2*] Patricia Pérez[3], Mar Ríos-Gutiérrez,[1] and María José Aurell[1]

[1] Department of Organic Chemistry, University of Valencia, Dr. Moliner 50, 46100 Burjassot, Valencia, Spain.

[2] Avd. Tirso de Molina 20, 46015, Valencia, Spain.

[3] Universidad San Sebastián, Facultad de Ciencias, Campus Ciudad Universitaria, Av. del Condor 720, Ciudad Empresarial, Huechuraba, Santiago, 8580704, Chile.

E-mail: luisrdomingo@gmail.com

Index

S2 Theoretical background of the Relative Interacting Atomic Energy (RIAE) analysis.

S3 **Figure S1** with the *ω*B97X-D/6-311+G(d,p) optimized geometries of the symmetric TSs.

S4 **Figure S2** with the plots of the NPA total atomic charge of the RCH_2^+ frameworks of the TSs of the series II and III of SN reactions with respect to those of the series I.

S5 **Figure S3** with the *ω*B97X-D/6-311+G(d,p) ELF basin attractor positions, populations of the C–X valence basins, and the sum of the population of the V(X) monosynaptic basins at the heteroatoms of the TSs associated with the symmetric series I of SN reactions of DMCs **3a-d**.

S6 **Figure S4** with the plot of the M06-2X/6-311+G(d,p) activation energies in DMSO of the symmetric series I of SN reactions of DMCs **3a-d** with chloride anion Cl^- with respect to the computed *ω*B97X-D/6-311+G(d,p) activation energies in DMSO.

S7 **Table S1** with the linear regression equations and Pearson correlation coefficient R^2 values from the plots of electrophilicity ω and nucleophilicity *N* indices of selected neutral molecules computed at the *ω*B97X-D/6-311+G(d,p) computational levels in DMSO vs those obtained at the B3LYP/6-31G(d) level in vacuo, and the Lower, upper and superior (super) limits of the electrophilicity and nucleophilicity *ω*B97X-D/6-311+G(d,p) scales in DMSO.

S7 **Table S2** with the *ω*B97X-D/6-311+G(d,p) electronic chemical potential μ, chemical hardness η, electrophilicity ω, and nucleophilicity *N* indices of the $CH_3OH_2^+$ and CH_3Cl species computed in DMSO.

S8 **Table S3** with the *ω*B97X-D/6-311+G(d,p) total electronic energies in DMSO of the stationary points involved in the three series of SN reactions of DMCs **1a–e** and **2a–e**.

S8 **Table S4.** M06-2X/6-311+G(d,p) total and relative electronic energies in DMSO of the stationary points involved in the series I of SN reactions of DMCs **1a–e**.

S9 *ω*B97X-D/6-311+G(d,p) computed total energies in DMSO, imaginary frequencies and Cartesian coordinates of the TS involved in SN reactions of DMC **1a–e** and **2a–e**.

S16 References.

Theoretical background of the Relative Interacting Atomic Energy (RIAE) analysis.

The Interacting Quantum Atoms[1] (IQA), based on the Quantum Theory of Atoms in Molecules[2,3] (QTAIM), divides the E_{total}^{IQA} total energy into two main energy contributions: the E_{intra}^{A} intra-atomic energies and the E_{inter}^{AB} interatomic energies (see Equation S1). The E_{inter}^{AB} energies are, in turn, divided into four additional electrostatic terms: the V_{ne}^{AB} and V_{en}^{AB} nuclei-electron interactions, the V_{ee}^{AB} electron-electron interactions, and the V_{nn}^{AB} nuclei-nuclei interactions (see Equation S3).

$$E_{total}^{IQA} = \sum E_{intra}^{A} + \sum E_{inter}^{AB} \quad \text{(S1)}$$

$$E_{intra}^{A} = T(A) + V_{ne}^{A} + V_{ee}^{A} \quad \text{(S2)}$$

$$E_{inter}^{AB} = ½V_{ne}^{AB} + ½V_{en}^{AB} + ½V_{ee}^{AB} + V_{nn}^{AB} \quad \text{(S3)}$$

Thanks to the additivity of the topological atoms,[4] an IQF approach has been recently introduced,[5] which allows the grouping of the IQA energy in terms of convenient fragments of the system. This enables a more chemically meaningful analysis of the interactions that take place between the atoms forming groups. In this sense, for Relative Interacting Atomic Energy[6] (RIAE) analysis of the nucleophilic substitution (SN) reactions, the atoms belonging to the molecular complexes (MCs) and the transition state structures (TSs) are regrouped into two interacting frameworks f(X), related to the DMC RCH_2Cl and the nucleophilic chlorine Cl reagents.

By default, the sum of all IQA atomic energies belonging to the considered framework f(X) (where X represents either the RCH_2Cl or Cl frameworks) at the TSs, and those of the corresponding MCs at the ground states, are computed. The RIAEs, i.e., the relative ξE_{total}^{X} total, ξE_{intra}^{X} intra-atomic, and ξE_{inter}^{X}interatomic energies, are obtained using Equations S4-S6. The symbol ξ denotes the IQA energy differences between the TS and the MC states of the two-interacting frameworks f(X); i.e. f(RCH_2Cl) and f(Cl) in the SN reactions.

$$\xi E_{total}^{X} = \xi E_{intra}^{X} + \xi E_{inter}^{X} \quad \text{(S4)}$$

$$\xi E_{intra}^{X} = \sum E_{intra}^{X(TS)} - \sum E_{intra}^{X(MC)} \quad \text{(S5)}$$

$$\xi E_{inter}^{X} = \sum E_{inter}^{X(TS)} - \sum E_{inter}^{X(MC)} \quad \text{(S6)}$$

The herein proposed RIAE analysis provides a measure of how much the two interacting frameworks f(X) are destabilized (resulting in positive relative energies) or stabilized (resulting in negative relative energies) when going from their MCs to the TSs. The sum of the ξE_{total}^{X} energies of the two interacting frameworks, $\xi E_{total}^{RCH2Cl+Cl}$,

provides the RIAE activation energy of the SN reactions obtained through the present EDA.[6-8]

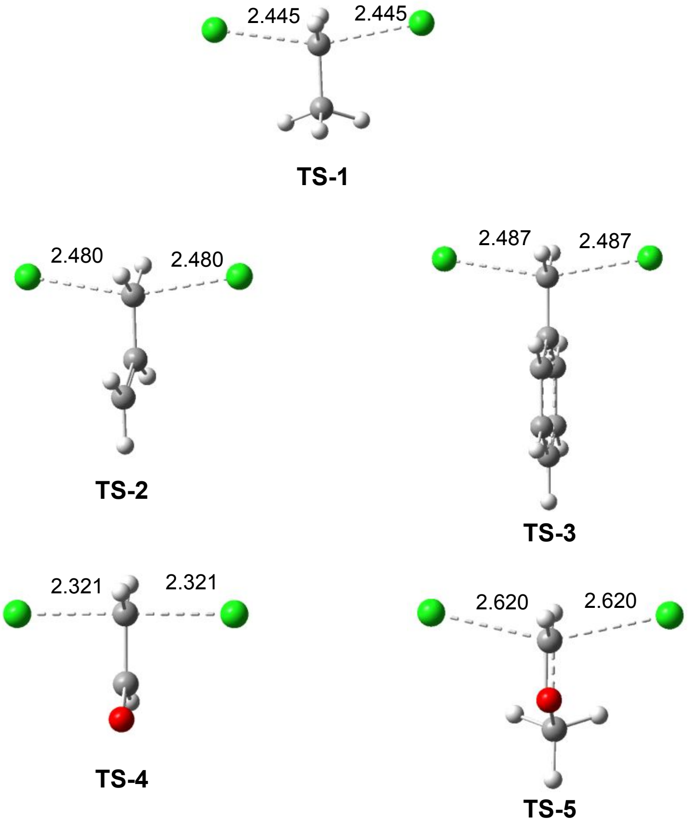

Figure S1. *ω*B97X-D/6-311+G(d,p) optimized geometries of the symmetric TSs. Distances are given in angstroms Å, while H–C–H bond angles are given degrees.

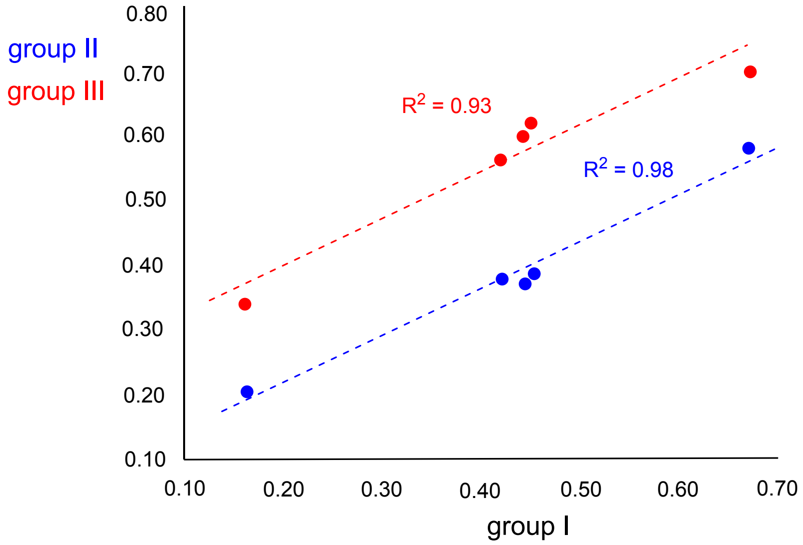

Figure S2. Plots of the NPA total atomic charge of the RCH_2^+ frameworks of the TSs of the SN reactions of series II and III with respect to those of series I. Charges are given as the average number of electrons, e.

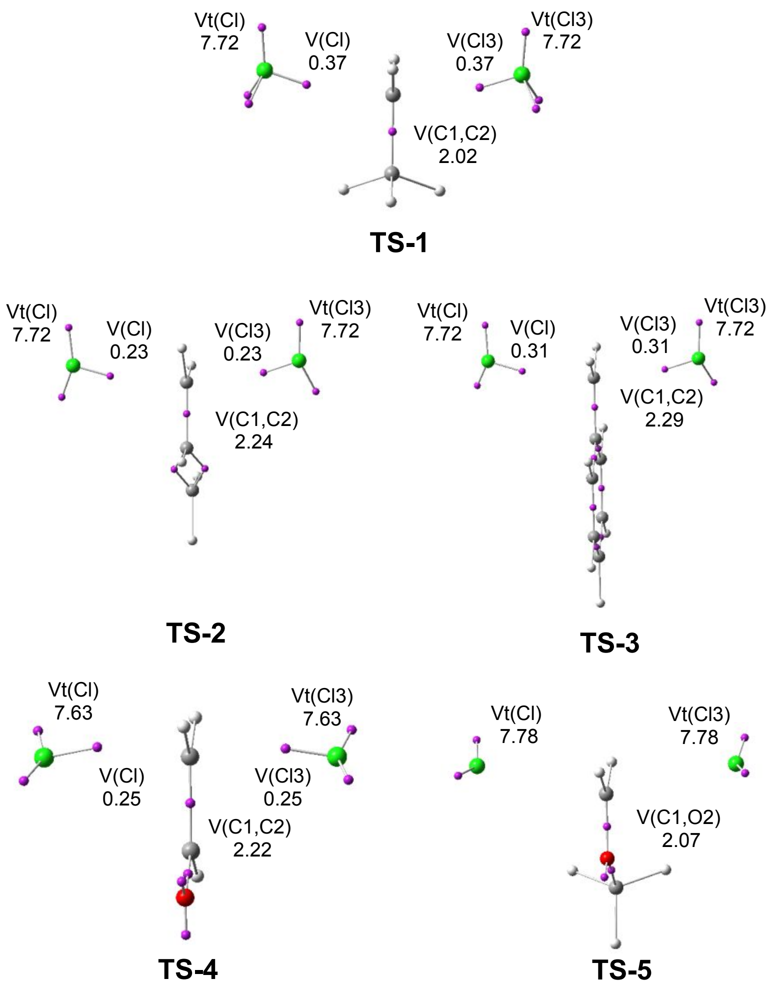

Figure S3. *ω*B97X-D/6-311+G(d,p) ELF basin attractor positions, populations of the C–X valence basins, and the sum of the population of the V(X) monosynaptic basins at the heteroatoms of the TSs associated with the symmetric series I of SN reactions of DMCs **3a-d**. Valence basin populations are given as the average number of electrons, e.

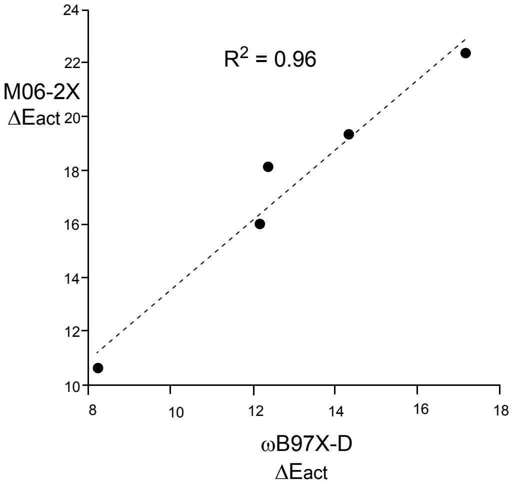

Figure S4. Plot of the M06-2X/6-311+G(d,p) activation energies in DMSO, ΔE_{act}, of the symmetric series I of SN reactions of DMCs **3a-d** with chloride anion Cl^- with respect to the computed *ω*B97X-D/6-311+G(d,p) activation energies in DMSO. Energies are expressed in $kcal \cdot mol^{-1}$.

Table S1. Linear regression equations and Pearson correlation coefficient R^2 values from the plots of electrophilicity ω and nucleophilicity *N* indices of selected neutral molecules given in refence 9 computed at the *ω*B97X-D/6-311+G(d,p) computational levels in DMSO vs those obtained at the B3LYP/6-31G(d) level in vacuo, and the Lower, upper and superior (super) limits of the electrophilicity and nucleophilicity *ω*B97X-D/6-311+G(d,p) scales in DMSO, in eV.

ω			*N*		
y = 0.4679x + 0.3774		R^2=0.99	y = 0.8876x – 0.2618		R^2=0.97
Lower	Upper	Super	Lower	Upper	Super
0.75	1.08	2.25	1.51	2.40	3.29

Table S2. *ω*B97X-D/6-311+G(d,p) electronic chemical potential μ, chemical hardness η, electrophilicity ω, and nucleophilicity *N* indices, in eV, of the CH_3-OH_2^+ and CH_3-Cl species computed in DMSO. The electrophilicity ω index of the CH_3-LG molecules corresponds with the nucleofugality Λ index of the LGs.

	LG	μ	η	ω	N
CH_3-OH_2^+	H_2O	–6.47	14.88	1.41	–2.83
CH_3-Cl	Cl^-	–4.38	12.41	0.77	0.50

Table S3. *ω*B97X-D/6-311+G(d,p) total electronic energies in DMSO, in a.u., of the stationary points involved in the three series of SN reactions of DMCs **1a–e** and **2a–e**.

**1a**	-539.436808	**2a**	-155.450489	**3a**	-253.551148
**1b**	-577.507774	**2b**	-193.519364	**3b**	-291.621861
**1c**	-731.148917	**2c**	-347.160304	**3c**	-445.263369
**1d**	-613.432832	**2d**	-229.444314	**3c**	-327.540077
**1e**	-614.641330	**2e**	-230.656384	**3e**	-328.751638
Cl^-	-460.393013	H_2O	-76.440243	Me_3N	-174.469667
**TS-1**	-999.792308	**TS-6**	-713.885607	**TS-11**	-615.831490
**TS-2**	-1037.869036	**TS-7**	-751.962849	**TS-12**	-653.906616
**TS-3**	-1191.512091	**TS-8**	-905.606782	**TS-13**	-807.549779
**TS-4**	-1073.796653	**TS-9**	-787.888106	**TS-14**	-689.826708
**TS-5**	-1075.017350	**TS-10**	-789.103707	**TS-15**	-691.054431

Table S4. M06-2X/6-311+G(d,p) total, E in a.u., and relative, ΔE in kcal·mol^{-1}, electronic energies computed in DMSO of the stationary points involved in the series I of SN reactions of DMCs **1a–e**.

	E	ΔE
**1a**	-539.402358	
**TS-1**	-999.745541	22.6
**1b**	-577.475016	
**TS-2**	-1037.823125	19.5
**1c**	-731.110652	
**TS-3**	-1191.460773	18.2
**1d**	-613.405332	
**TS-4**	-1073.758876	16.1
**1e**	-614.608257	
**TS-5**	-1074.970474	10.6

ωB97X-D/6-311+G(d,p) computed total energies in DMSO, imaginary frequencies and Cartesian coordinates of the TS involved in SN reactions of DMC **1a–e** and **2a–e**.

TS-1
E(RwB97XD) = -999.792308433 A.U.
FREQ = -451.7100 cm-1

6	-0.000299	-0.012453	0.113697
1	-0.000127	-0.944438	-0.425067
1	-0.000274	0.896726	-0.457783
17	-2.407746	0.048949	-0.309602
17	2.407746	0.049632	-0.308991
6	0.000313	-0.009828	1.605110
1	0.877481	-0.545000	1.970164
1	0.004777	1.003768	2.004193
1	-0.881258	-0.537302	1.970788

TS-2
E(RwB97XD) = -1037.86903634 A.U.
FREQ = -410.4205 cm-1

6	0.000584	0.000006	-0.000082
6	0.003253	-0.000106	1.333785
1	0.927575	0.000524	-0.565138
1	-0.925800	-0.000425	-0.562213
1	-0.925953	-0.000636	1.893079
6	1.233074	0.000445	2.100962
1	1.217408	0.000251	3.174633
1	2.187722	0.001050	1.601199
17	1.525485	2.445643	2.392179
17	1.528222	-2.444531	2.391329

TS-3
E(RwB97XD) = -1191.51209095 A.U.
FREQ = -397.8481 cm-1

6	0.000050	0.000652	0.000841
6	0.000310	0.000842	1.387166
6	1.209963	0.000111	2.089642
6	2.418534	-0.000803	1.385311
6	2.416671	-0.000968	-0.001013
6	1.207828	-0.000247	-0.693542
1	-0.938282	0.001200	-0.541197
1	-0.935726	0.001534	1.935092
1	3.355407	-0.001354	1.931805
1	3.354167	-0.001654	-0.544495
1	1.206993	-0.000385	-1.777668
6	1.211088	0.000300	3.538428
1	2.136209	-0.000551	4.087279
1	0.286826	0.001280	4.088719

17	1.208986	-2.449562	3.966710
17	1.213853	2.450265	3.966094

TS-4
E(RwB97XD) = -1073.79665307 A.U.
FREQ = -557.9478 cm-1

6	-1.274953	2.111175	-0.515957
8	-0.299318	2.332568	0.158434
6	-2.047714	0.835330	-0.453951
1	-2.909826	0.694544	-1.080237
1	-1.734590	0.057299	0.223129
17	-0.746972	-0.016923	-2.176601
1	-1.667239	2.849063	-1.235039
17	-3.448637	1.778221	1.138255

TS-5
E(RwB97XD) = -1075.01734967 A.U.
FREQ = -197.1631 cm-1

6	0.000000	0.000000	0.000000
1	0.000000	0.000000	1.078024
1	0.914608	0.000000	-0.572911
17	0.560396	2.555780	0.132221
17	0.560513	-2.555767	0.132375
8	-1.136534	-0.000039	-0.567514
6	-1.144761	-0.000074	-2.012520
1	-0.641199	-0.901502	-2.362237
1	-2.189418	0.000131	-2.306710
1	-0.640830	0.901126	-2.362296

TS-6
E(RwB97XD) = -713.885606594 A.U.
FREQ = -531.4785 cm-1

6	0.038518	-0.256144	0.233517
1	0.959549	-0.041984	-0.280416
1	-0.631586	-0.977841	-0.200805
7	1.149811	-2.085356	0.833659
17	-0.967326	1.346934	-1.079264
6	2.368256	-1.717746	1.548980
6	1.462207	-2.702548	-0.452945
6	0.293924	-2.952982	1.637385
1	2.022457	-3.637847	-0.323187
1	0.537577	-2.922177	-0.990458
1	2.062927	-2.017243	-1.054277
1	0.038123	-2.459369	2.576695
1	-0.627201	-3.166016	1.091416
1	0.793429	-3.903185	1.870558
1	2.115214	-1.227958	2.491028

1	2.983075	-2.600195	1.772502
1	2.955159	-1.026148	0.941251
6	-0.248465	0.344455	1.573041
1	-1.322264	0.440148	1.728987
1	0.147752	-0.285454	2.371181
1	0.215978	1.325701	1.662595

TS-7
E(RwB97XD) = -751.962848967 A.U.
FREQ = -475.3707 cm-1

6	-0.003074	-0.000484	0.000870
6	0.001384	0.001502	1.333028
1	0.921954	-0.002774	-0.567334
1	-0.931081	-0.007241	-0.558532
1	-0.929741	-0.010363	1.889666
6	1.230487	0.036004	2.115975
1	1.195945	0.172293	3.181901
7	1.441809	2.294559	2.160080
6	1.789680	2.774092	0.829830
1	2.709334	2.291074	0.492731
1	0.986714	2.528673	0.131965
1	1.940622	3.862649	0.822302
6	2.529425	2.505815	3.109423
1	2.759719	3.573643	3.226735
1	2.251340	2.102638	4.085436
1	3.427049	1.991069	2.760419
6	0.192940	2.882305	2.631439
1	-0.078418	2.445696	3.595008
1	0.281919	3.971320	2.750234
1	-0.601834	2.672171	1.913622
1	2.188296	0.067059	1.621661
17	1.499910	-2.220278	2.522083

TS-8
E(RwB97XD) = -905.606781677 A.U.
FREQ = -465.7898 cm-1

6	0.002574	0.003856	-0.004378
6	0.000808	-0.004818	1.383011
6	1.207216	-0.005722	2.088637
6	2.414864	0.012628	1.385467
6	2.415557	0.020755	-0.002085
6	1.209750	0.015083	-0.698182
1	-0.936022	0.002174	-0.546124
1	-0.937439	-0.010562	1.927173
1	3.352088	0.021796	1.931341
1	3.355117	0.032476	-0.542032
1	1.210750	0.021801	-1.782213
6	1.205430	-0.049794	3.546142
1	2.131950	-0.125861	4.089401

1	0.278590	-0.144853	4.085680
17	1.181155	2.216328	4.041756
7	1.232179	-2.313046	3.750228
6	1.177204	-2.504727	5.196041
1	0.259818	-2.064359	5.591998
1	1.195789	-3.570805	5.460931
1	2.032365	-2.014708	5.666030
6	0.066019	-2.886095	3.089782
1	0.013638	-3.973182	3.242888
1	-0.843406	-2.432722	3.489876
1	0.115773	-2.684563	2.018423
6	2.472771	-2.832754	3.188620
1	2.503849	-2.627344	2.117231
1	3.325654	-2.344137	3.664625
1	2.558539	-3.917459	3.343016

TS-9
E(RwB97XD) = -787.888105880 A.U.
FREQ = -546.3827 cm-1

6	1.354044	-0.072384	2.186072
6	1.089496	-0.108528	3.651924
1	1.914139	-0.116416	4.344633
1	0.076344	-0.207281	4.007841
17	1.039817	2.106687	3.683352
7	1.200524	-2.261128	3.738429
6	1.265010	-2.505467	5.180866
1	0.396020	-2.060532	5.668588
1	1.278164	-3.581361	5.393639
1	2.171592	-2.057823	5.591758
6	-0.011885	-2.846569	3.163736
1	-0.011416	-3.937172	3.285034
1	-0.888950	-2.437324	3.667850
1	-0.069935	-2.604184	2.102810
6	2.403972	-2.765725	3.071858
1	2.329221	-2.599330	1.996357
1	3.281819	-2.242640	3.455305
1	2.527186	-3.841205	3.250300
1	2.400430	0.133052	1.906841
8	0.501485	-0.268291	1.354480

TS-10
E(RwB97XD) = -789.103706524 A.U.
FREQ = -327.7479 cm-1

6	2.528183	0.171407	1.461711
1	3.295278	-0.375824	2.010743
6	1.158263	-0.013102	3.332370
1	2.047935	-0.030152	3.944173
1	0.176429	-0.143862	3.754058
17	1.032414	2.322742	3.806039

7	1.240303	-2.391234	3.792620
6	1.254654	-2.560265	5.238516
1	0.385062	-2.064497	5.676358
1	1.232834	-3.621901	5.530067
1	2.157902	-2.107223	5.653722
6	0.022668	-2.922487	3.197249
1	-0.053283	-4.014074	3.319809
1	-0.849876	-2.462031	3.666519
1	0.006751	-2.690033	2.129870
6	2.425007	-2.965356	3.170957
1	2.403871	-2.775237	2.095327
1	3.323796	-2.508629	3.591861
1	2.483975	-4.054175	3.326260
1	2.466446	-0.182606	0.436819
1	2.723993	1.243717	1.490565
8	1.235977	-0.066420	2.049201

TS-11
E(RwB97XD) = -615.831489861 A.U.
FREQ = -423.3138 cm-1

6	-0.578532	-0.122317	-0.075806
1	-0.506380	0.819229	-0.595793
1	-0.541579	-0.101646	1.000449
17	1.919068	0.323613	0.285362
8	-2.561893	0.069192	-0.062622
1	-2.985131	-0.639068	0.436192
1	-2.816002	0.889633	0.375019
6	-0.531927	-1.399749	-0.831012
1	0.499779	-1.587434	-1.128579
1	-0.872341	-2.237118	-0.222432
1	-1.131144	-1.320077	-1.738411

TS-12
E(RwB97XD) = -653.906615796 A.U.
FREQ = -372.2658 cm-1

6	-0.025595	0.011109	-0.046797
6	-0.090026	0.053423	1.286268
1	0.924541	-0.079533	-0.564097
1	-0.919678	0.074913	-0.655446
1	-1.038150	0.150621	1.802191
6	1.105415	-0.048363	2.096116
1	1.068112	0.108789	3.161146
1	2.078560	-0.028523	1.628824
8	1.265547	-1.983464	2.392778
1	1.128527	-2.455051	1.562133
1	0.580294	-2.298303	2.994270
17	1.580509	2.592414	2.351373

TS-13
E(RwB97XD) = -807.549779008 A.U.
FREQ = -331.2383 cm-1

6	1.029615	-0.099818	2.088384
1	0.979640	0.077761	3.150895
1	2.003103	-0.075926	1.623376
8	1.102984	-2.042020	2.421341
1	1.949171	-2.300542	2.807138
1	1.028198	-2.522413	1.587937
17	1.652413	2.565614	2.234473
6	-0.160816	-0.008656	1.280016
6	-1.414712	0.119385	1.891559
6	-0.065666	-0.066571	-0.116722
6	-2.558037	0.193076	1.113649
1	-1.481542	0.165706	2.972681
6	-1.212249	0.006282	-0.890896
1	0.907826	-0.161176	-0.585459
6	-2.455041	0.136582	-0.275005
1	-3.528793	0.296386	1.582857
1	-1.142217	-0.034750	-1.970964
1	-3.351059	0.195943	-0.882220

TS-14
E(RwB97XD) = -689.826707801 A.U.
FREQ = -496.4085 cm-1

6	-0.016130	0.113588	1.349422
1	-0.906107	0.492261	1.869786
6	1.202231	-0.097495	2.209864
1	1.137317	0.041016	3.278275
1	2.174405	-0.064575	1.742576
8	1.210221	-1.918573	2.308479
1	1.159429	-2.329936	1.435287
1	0.494511	-2.288089	2.842215
17	1.215974	2.353143	2.085475
8	-0.022366	-0.196251	0.183688

TS-15
E(RwB97XD) = -691.054431963 A.U.
FREQ = -98.7853 cm-1

8	0.000000	0.000000	0.000000
6	0.000000	0.000000	1.293160
8	1.667017	0.000000	1.877208
17	-3.093245	0.000000	1.706994
1	-0.251654	-0.955118	1.733112
1	-0.311956	0.894386	1.824419
1	2.106905	0.860461	1.870916
1	2.221894	-0.617975	1.381406
6	0.025270	1.276178	-0.674954

1	-0.333703	1.087083	-1.682125
1	-0.634150	1.975781	-0.160077
1	1.048570	1.649832	-0.704168

References

1. Blanco, M. A.; Martín Pendás, A.; Francisco, E. Interacting Quantum Atoms: A Correlated Energy Decomposition Scheme Based on the Quantum Theory of Atoms in Molecules, *J. Chem. Theory Comput.* **2005**, *1*, 1096–1109.
2. Bader, R.F.W.; Tang, Y.H.; Tal, Y.; Biegler-König, F.W. Properties of atoms and bonds in hydrocarbon molecules. *J. Am. Chem. Soc*. **1982**, *104*, 946–952.
3. Bader, R.F.W. *Atoms in Molecules: A Quantum Theory*, Oxford University Press, Oxford, New York, 1994.
4. Martín Pendás, A.; Blanco, M.A.; Francisco, E. Chemical Fragments in Realpace: Definitions, Properties and Energetic Decompositions. *J. Comput. Chem.* 2007, **28**, 161–184.
5. Triestram, L.; Falcioni, F.; Popelier, P. L. A. Interacting Quantum Atoms and Multipolar Electrostatic Study of XH···π Interactions, *ACS Omega* **2023,** *8*, 34844–34851.
6. Domingo, L.R.: Ríos-Gutiérrez, M.; Pérez, P.; Understanding the Electronic Effects of Lewis Acid Catalysts in Accelerating Polar Diels-Alder Reactions. *J. Org. Chem.* **2024**. 89, 12349-12359.
7. Domingo, L.R.; Pérez, P.; Ríos-Gutiérrez, M.; Aurell, M.J. A Molecular Electron Density Theory Study of Hydrogen Bond Catalysed Polar Diels–Alder Reactions of α,β-unsaturated Carbonyl Compounds, *Tetrahedron Chem.* **2024**, *10*,100064.
8. Domingo, L.R.; Ríos-Gutiérrez, Revealing the Decisive Role of Global Electron Density Transfer in the Reaction Rate of Polar Organic Reactions within Molecular Electron Density Theory, *Molecules* **2024**, *29*, 1.
9. Domingo, L.R.; Ríos-Gutiérrez, M.; Pérez, P. Electrophilicity ω and Nucleophilicity N Scales for Cationic and Anionic Species, *Sci. Rad.* **2025**, *4*, 1-17.
